# Margaret M. Stark, ed. Clinical Forensic Medicine. A Physician's Guide

**DOI:** 10.3325/cmj.2021.62.537

**Published:** 2021-10

**Authors:** Vedrana Petrovečki

**Field of medicine:** Forensic medicine, emergency medicine, surgery

**Format:** Hardcover book, e-book

**Audience:** This book covers wide areas of medicine, with potential diverse readership. The target audience are forensic specialists; however, the book will be of interest to emergency medicine specialists, surgeons, pediatricians, gynecologists, family physicians, and others. It might also be interesting to toxicologists, prosecutors, judges, and criminal lawyers.

**Purpose:** Although the term clinical forensic medicine has been used since the middle of the last century, it is still almost unknown in Croatia. Croatian physicians practice clinical forensic medicine without being aware of it. All physicians involved in the identification, description, and care of injuries related to a criminal offense committed on living persons are in fact engaged in clinical forensic medicine. As the book reveals to a careful reader, every physician needs to possess a basic knowledge of forensic medicine, but also of clinical forensic medicine.

**Content:** The book consists of 14 chapters. The first chapter outlines the history of clinical forensic medicine and defines relevant terms related to the field. Although clinical forensic medicine is widely used, the education and practice of the discipline are not regulated by any international standards. The importance of this area of medicine is shown by the establishing of the Faculty of Clinical Forensic Medicine in 2014 by the Royal College of Pathologists of Australasia (https://www.rcpa.edu.au/Trainees/Faculties/FCFM).

The second chapter is dedicated to the ethical principles and legal regulations applied in clinical forensic medicine. Since the chapter is based on English and Welsh law, it is not practically useful to Croatian readers, but can serve for comparison with other legislative frameworks.

The third and the most comprehensive chapter is devoted to sexual assault and the examination necessary to confirm this type of assault. The chapter covers all aspects of determining sexual abuse, beginning with the description of the physiology and anatomy of genital organs and the anorectal area. The chapter describes in detail the examination procedure and sampling, and the procedures performed to detect and record injuries acquired during unwanted penetration in women, men, and children. The chapter also lists the procedures required to prevent pregnancy and sexually transmitted diseases.

For anyone involved in clinical forensics, the forth chapter is the book's most important chapter (in my opinion, because of the significance of the addressed issues, this chapter should precede the third chapter). The chapter discusses injury assessment, documentation, and interpretation. Each part of the examination is described in detail – from taking the medical history and status, potential limiting circumstances during the examination, to the instructions on how to record the identified injuries. A part of this chapter is dedicated to the basic knowledge about the most common injuries encountered in clinical forensic medicine – mechanical injuries.

The fifth chapter also deserves special attention. In light of the increasing identification of injuries raising the suspicion of child physical abuse, this chapter can be interesting to pediatricians, general practitioners, and family physicians, but also to all experts working with children. The chapter acquaints the reader with the epidemiology and risk factors for physical abuse. The procedures to be taken in the case of suspected abuse are described, as well as possible consequences of abuse. Special attention is paid to the most common injuries encountered in child physical abuse. A part of this chapter is also devoted to fabricated or induced illness, previously known as “Munchausen Syndrome by Proxy,” a disorder in which parents or guardians make up or cause the child's symptoms.

The book also includes chapters dedicated to the consequences of the use of chemical agents for crowd control (chapter 6), injuries resulting from restraint during arrest (chapter 7), and injuries resulting from the use of conducted electrical weapons (electroshock weapons, chapter 8). Three chapters deal with different problems encountered in the population of detainees. Chapter 9 discusses the treatment of detainees suffering from chronic physical and mental diseases; chapter 10 describes the most common infectious diseases in this population and their treatment options, as well as the protection of detainees' contacts; and chapter 11 discusses the topic of detainees’ fitness to be questioned and convicted of a crime. The chapter lists diseases and conditions that may temporarily or permanently prevent this fitness. Chapter 12 is of particular interest to toxicologists, discussing the abuse of drugs and addictive substances (alcohol, drugs), as well as their impact on the commission of criminal offenses. Chapter 13 is devoted to deaths in police custody, which require an autopsy and expertise of a forensic pathologist to exclude a violent death. A separate chapter (chapter 14) deals with the impact of addiction on driving ability, and includes a brief description of physical illnesses that limit this ability.

**Highlights:** The book is well-organized, with uniformly structured chapters, each containing an introduction with learning objectives, key points, and self-assessment exercises. At the end of each chapter, the reader can find an extensive reference list. The index at the end of the book facilitates finding the topics of interest. The relative absence of images is justified by the fact that this is not a forensic medicine textbook, but a (detailed) guide enabling physicians to apply their knowledge from forensic medicine into clinical practice.

**Commentary:** This systematically written book gradually introduces the reader to a field of medicine that involves collaboration between the physicians, police, and legal system. It covers the wide area of clinical forensic medicine. The chapters on ill-treatment and death during apprehension and infectious diseases seem almost prophetic, as at the time of writing, no one could have thought that in 2020 the world will be faced with the brutal death of George Floyd and the SARS-CoV-2 pandemic. Including the issues related to human and organ trafficking as well as migration might represent a valuable addition to the next edition.

